# Quantitative proteomic analysis reveals the effects of mu opioid agonists on HT22 cells

**DOI:** 10.3389/fphar.2022.1022449

**Published:** 2023-01-09

**Authors:** Xutong Zhang, Yani Lou, Dongxu Zheng, Jialin Lu, Dansi Qi

**Affiliations:** ^1^ Department of Anesthesiology, The Second Affiliated Hospital and Yuying Children’s Hospital of Wenzhou Medical University, Wenzhou, China; ^2^ Department of Pathology, Second Affiliated Hospital and Yuying Children’s Hospital of Wenzhou Medical University, Wenzhou, China

**Keywords:** mu opioid agonist, proteomic, loperamide, HT22 cell, nerve injury, LC-MS/MS

## Abstract

**Introduction:** At present, the mu opioid receptor is the most important neuroaesthetics receptor in anesthesiology research, and the damage that it does to the nervous system is unknown.

**Methods:** We investigated the effects of loperamide, an agonist of the mu opioid receptor, on protein expression in HT22 cells using stable isotope labeling of amino acids in cell culture (SILAC), immobilized metal affinity chromatography (IMAC) enrichment, and high-resolution liquid chromatography-tandem mass spectrometry (LC-MS/MS). A total of 7,823 proteins were identified.

**Results and Discussion:** Bioinformatic analysis revealed that mu opioid receptor agonism can induce distinct changes in the proteome of HT22 cells. These findings improve our understanding of narcotic drugs.

## Introduction

In the opioid system, mu, delta, and kappa receptors are G protein-coupled receptors that are stimulated by endogenous opioid peptides (Kieffer, 1995). Opioid receptors can also be exogenously activated by alkaloid opioids, the prototypical example of which is morphine. In mice lacking mu opioid receptors, morphine’s addictive and analgesic characteristics are eliminated, proving that mu receptors are responsible for both the drug’s beneficial and harmful effects (Matthes et al., 1996). The potentiating effects of alcohol, cannabis, and nicotine all work on various receptors and are significantly decreased in these mutant mice, according to many studies (Kieffer and Gaveriaux-Ruff, 2002). Therefore, mu opioid receptor activation has important clinical research significance in nervous system injury (Tiwari et al., 2018). Expanding our understanding of mu receptor function will be of great help as we learn more about the general mechanisms of nerve injury. After direct (morphine) or indirect stimulation (alcohol, cannabinoid, nicotine) of the mu opioid receptors, positive reinforcement occurs, so an understanding of mu receptor function is critical for addiction treatment and the development of neurological damage. A recent study in neurons indicates that mu receptor signaling and modulation are strongly dependent on agonist action. Loperamide is a potent mu opioid agonist which is routinely used to treat diarrhea and as an adjunctive analgesic for oral pain due to mucositis or cancer (Regnard et al., 2011). As loperamide is an effective mu opioid receptor agonist, we used HT22 cells to study the effect of mu opioid receptor agonists on neurons nerve injury and to investigate mu opioid receptor agonists. Actions of opioid receptors. We explored the expression of related proteins in HT22 cells.

Among the brain’s most important parts are the hippocampus and it is responsible for the integration of information between short-term and long-term memory. General anesthesia is known to involve the hippocampus, and therefore, there are concerns that it may inhibit learning and memory. For the research of Alzheimer’s disease, Parkinson’s disease, Kinson’s disease, and the effects of general anesthesia, HT22 cells are frequently employed as a neurobiological cell model. They are produced from HT4 cells, which were initially immortalized from primary mouse hippocampus neuronal cultures (Behl et al., 1995; Behl, 1998; Kulich and Chu, 2001; Schafer et al., 2004; Zhu et al., 2014). Chronic pain is neuropathic for approximately 25%–30% of patients and affects 7%–8% of adults in the general population (Torrance et al., 2014). These individuals frequently endure unpredictable, paroxysmal, and difficult-to-treat pain. However, evoked reflex withdrawal reactions to external stimuli are frequently used to infer neuropathic pain-related behaviors in animal models. Clinically relevant behaviors that are suggestive of persistent pain are more complex and less studied pre-clinically. Therefore, we took a novel approach to examine the proteomic changes caused by the activation of mu opioid receptors in nerve cell injury, to identify relevant clinical markers.

The LC-MS/MS technique is a high-throughput, high-precision proteomic analysis technique that has become a powerful method for analyzing protein expression changes across a variety of species (Edmondson et al., 2002; Bouwmeester et al., 2004; Herring et al., 2015). In this study, we comprehensively compared the proteome of HT22 cells treated with the mu opioid receptor agonist loperamide using an LC-MS/MS-based method. The goal of this study was to reveal the influences of mu opioid receptor activation on proteins related signal transduction in nerve cells, which may help us understand the mechanism of mu opioid receptors in nervous system damage.

## Materials and methods

### Labeling and drug treatment of HT22 cells

Based on the instructions provided by the manufacturer, HT22 cells were labeled using the Stable Isotope Labeling of Amino Acids in Cell Culture (SILAC) protein quantification kit (Pierce, Thermo Fisher Scientific). Cells were treated with 3.8948 μg/mL loperamide for 8 h and then washed twice with ice-cold phosphate-buffered saline (PBS) and harvested.

### Trypsin digestion and fractionation

The solution was digested at 37°C for 16 h with trypsin (Promega) at a ratio of 50:50 (w/w). A final concentration of 5 mM of dithiothreitol (DTT) was added, followed by a 1-h incubation period at 37°C. Incubation at room temperature for 30 min in the dark with iodoacetamide (IAA) at a final concentration of 15 mM was then carried out to alkylate proteins. Adjusting the solution’s final cysteine content to 30 mM and letting it sit at room temperature for 30 min stopped the alkylation reaction. Final trypsin: protein ratio of 1:100 (w/w) was added to the solution and incubated at 37°C for 4 h to complete digestion. High pH reverse-phase high-performance liquid chromatography (HPLC) was used to separate the sample using an Agilent 300 Extend C18 column with 5 m particles that had an ID of 4.6 mm and a length of 250 mm. Peptides were initially separated into 80 fractions over 80 min using a gradient of 2%–60% acetonitrile in 10 mM ammonium bicarbonate (pH 10). Following that, the peptides were mixed into 18 fractions and dried using vacuum centrifugation.

### IMAC enrichment of phosphopeptides

An immobilized metal affinity chromatography (IMAC) microsphere suspension was first added to the peptide mixtures and incubated under vibration. After centrifugation, phosphopeptide-enriched IMAC microspheres were obtained with the supernatant removed. To remove non-specifically adsorbed peptides from IMAC microspheres, 50% acetonitrile (ACN)/6% trifluoroacetic acid (TFA) was washed sequentially with 30% ACN/0.1% TFA. IMAC microspheres were used to elute the enriched phosphopeptides using an elution buffer containing 10% NH_4_OH. For LC-MS/MS analysis, the phosphopeptide-containing supernatant was gathered and lyophilized.

### LC-MS/MS analysis

After being dissolved in solvent A [.1% formic acid (FA) in 2% ACN], the peptides were immediately loaded onto a reverse-phase pre-column (Acclaim PepMap 100, Thermo Fisher Scientific). On an EASY-LLC 1000 UPLC system, peptides were separated using a reverse-phase analytical column (Thermo Fisher Scientific, Acclaim PepMap RSLC) with a linear gradient of 4%–22% solvent B (.1% FA in 98% ACN) for 50 min, 22%–35% solvent B for 12 min, 35%–85% solvent B for 4 min, and then 85% solvent B for 3 min. Using a Q Exactive Plus hybrid quadrupole-Orbitrap mass spectrometer, the resultant peptides were examined (Thermo Fisher Scientific).

Peptides were submitted to tandem mass spectrometry (MS) in Q Exactive Plus (Thermo Fisher Scientific) connected to an ultra-performance liquid chromatograph after nanospray ionization (NSI).At a resolution of 70,000, Orbitrap was used to detect intact peptides. The normalized collision energy (NCE) of 30 was used to select peptides for MS/MS analysis and ion fragments were detected using Orbitrap at a resolution of 17,500. Data-dependent procedures were applied to the top 20 precursor ions, alternating between one MS scan and 20 MS/MS scans, with a dynamic exclusion period of 15.0 s. The electrospray voltage that was used was 2.0 kV. Automatic gain control (AGC) targets of 3 106 ions and a maximum injection time of 50 m were used to collect MS1 spectra, whereas an AGC target of 5 104 ions and a maximum injection duration of 200 ms were used to acquire MS2 spectra. The m/z scan range for the MS scans was 350–1,800. Using the dataset identifier PXD004687, mass spectrometry and proteomics data were deposited with the ProteomeXchange Consortium *via* the PRIDE50 partner repository.

### Database search and bioinformatics analysis

The obtained MS/MS data were processed using MaxQuant (v.1.4.1.2), which has an integrated Andromeda search engine. Combining the SwissProt_mouse database with a reverse decoy database, tandem mass spectra were searched. Trypsin/P was defined as cleavage enzyme, and up to two missed cleavages, five modifications per peptide, and five charges were allowed. Ion precursors have a mass error of 10 ppm, while fragments have a mass error of .02 Da. Met oxidation and acetylation were designated as variable modifications, but Cys carbamidomethylation was designated as a permanent modification. Ser, Thr, and Tyr phosphorylations were also designated as variable modifications for the phosphoproteome investigation. The thresholds for false discovery rate (FDR) for proteins, peptides, and modification sites were established at 1%. Seven residues were chosen as the bare minimum for peptide length. To be employed in the next analysis, the other parameters in MaxQuant were set to larger than .75.

The Database for Annotation, Visualization, and Integrated Discovery was used to analyze GO term association and enrichment. Using DAVID’s Functional Annotation Tool, domain analysis was carried out on the InterPro database. Protein complexes were analyzed using the hand-maintained CORUM protein complex database. Using Cytoscape, the STRING database system was used to display functional protein-protein interaction networks. A bioinformatics study was conducted, and significant results were defined as adjusted *p*-values < .05.

### Cell growth assay

Measurement of cell growth was done using the CCK8 kit based on the directions provided by the manufacturer (Solarbio, #CA1210). A 5,000-cell density was used in 96-well plates, and loperamide was applied for 8 h to HT22 cells. The cells were then given 10 L of CCK8 to each well, and they were left to react for 4 h. On a plate reader (Heales, #MB-580), the optical densities of the medium in the wells were determined at 450 nm.

### RNA isolation and quantitative reverse transcriptase PCR

Using the TRIzol^®^ Plus RNA Purification Kit (Invitrogen, #12183-555), RNA was isolated from HT22 cells in accordance with the manufacturer’s instructions. The Loperamide HCl treatment group was used as the experimental group, and the DMSO treatment group was used as the control. A quantitative real-time polymerase chain reaction (RT-PCR) was performed on a StepOnePlus Real-Time PCR System ABI using the One-Step RT-qPCR Kit (Sangon Biotech, B639277). Primer sequences are listed in Supplementary Table S1 and expression levels were normalized to glyceraldehyde-3-phosphate dehydrogenase (GAPDH) was used as the reference for relative quantification.

### Statistics

Data are presented as the mean ± standard error of the mean (SEM) and were gathered from at least three different experiments. Using the Student’s t-test, statistical significance between the two groups was analyzed. *p*-values < .05 were used to determine statistical significance for differences. The statistical program GraphPad Prism (GraphPad Software Inc., La Jolla, CA, United States) was used to conduct all tests.

## Results

### Strategies for quantitative proteome analysis

**FIGURE 1:**
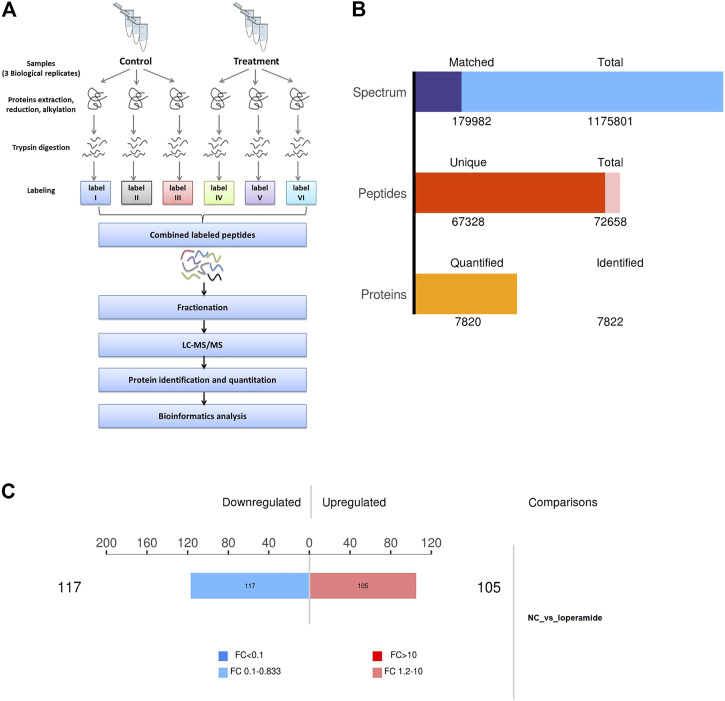
Overview of the proteomes of loperamide-treated HT22 cells. **(A)** Systematic workflow of the quantitative proteoome analyses. **(B)** Statistical histogram of identification and quantification results. **(C)** Histogram of differential protein quantification results.

In this study, we combined SILAC labeling methods, HPLC fractionation, IMAC enrichment, Orbitrap mass spectrometry with high resolution, and a bioinformatics analytic system for quantification. The workflow for the proteome analysis of control and loperamide-treated HT22 cells is shown in Figure 1A. Using this method, we identified 7,820 proteins in HT22 cells (Figure 1B) and a total of 222 differentially expressed proteins were identified in the proteomic analysis of loperamide-treated cells (Figure 1C).

### Effects of loperamide on the overall proteome of HT22 cells

Using thresholds of fold-change >1.2 and *p*-value <.05 (*t*-test), In HT22 cells treated with loperamide, we found that 105 proteins were upregulated and 117 proteins were downregulated (Supplementary Table S1).

**FIGURE 2:**
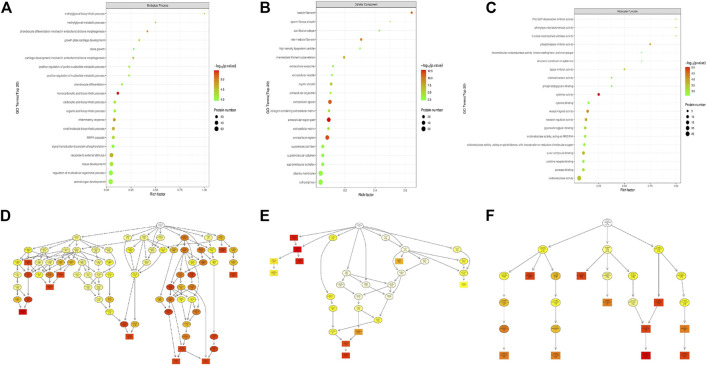
KEGG pathway enrichment analysis of differentially expressed proteins in loperamide-treated HT22 cells. **(A)** KEGG pathway annotation statistics for differentially expressed proteins. **(B)** KEGG pathway differential expression analysis map. **(C)** KEGG pathway enrichment bubble map.

Differentially expressed proteins in the loperamide-treated HT22 cells were analyzed for GO and KEGG enrichment (Figure 2). In the biological process category, loperamide-treated cells showed changes in proteins involved in monocarboxylic acid biosynthetic process, chondrocyte differentiation involved in endochondral bone morphogenesis, inflammatory response, response to external stimuli, methylglyoxal biosynthesis process, among others (Figure 2A). In the cytokine activity category, receptor ligands and molecular functions such as activity, phospholipase inhibitor activity, receptor modulator activity, and sulfur compound binding, were identified, among others (Figure 2B). Additionally, we identified changes in proteins categorized as having functions involving the localization of the extracellular domain, extracellular domain, extracellular space, keratin filament, and intermediate filament, among others (Figure 2C). To show the hierarchical relationship of the enriched GO entries of differential proteins, top GO-directed acyclic graph (DAG) was used to display the top-down defined functional scope, in which the branch represents the inclusion relationship and the lower the branch represents the more specific the defined functional scope is (Figures 2D–F).

**FIGURE 3:**
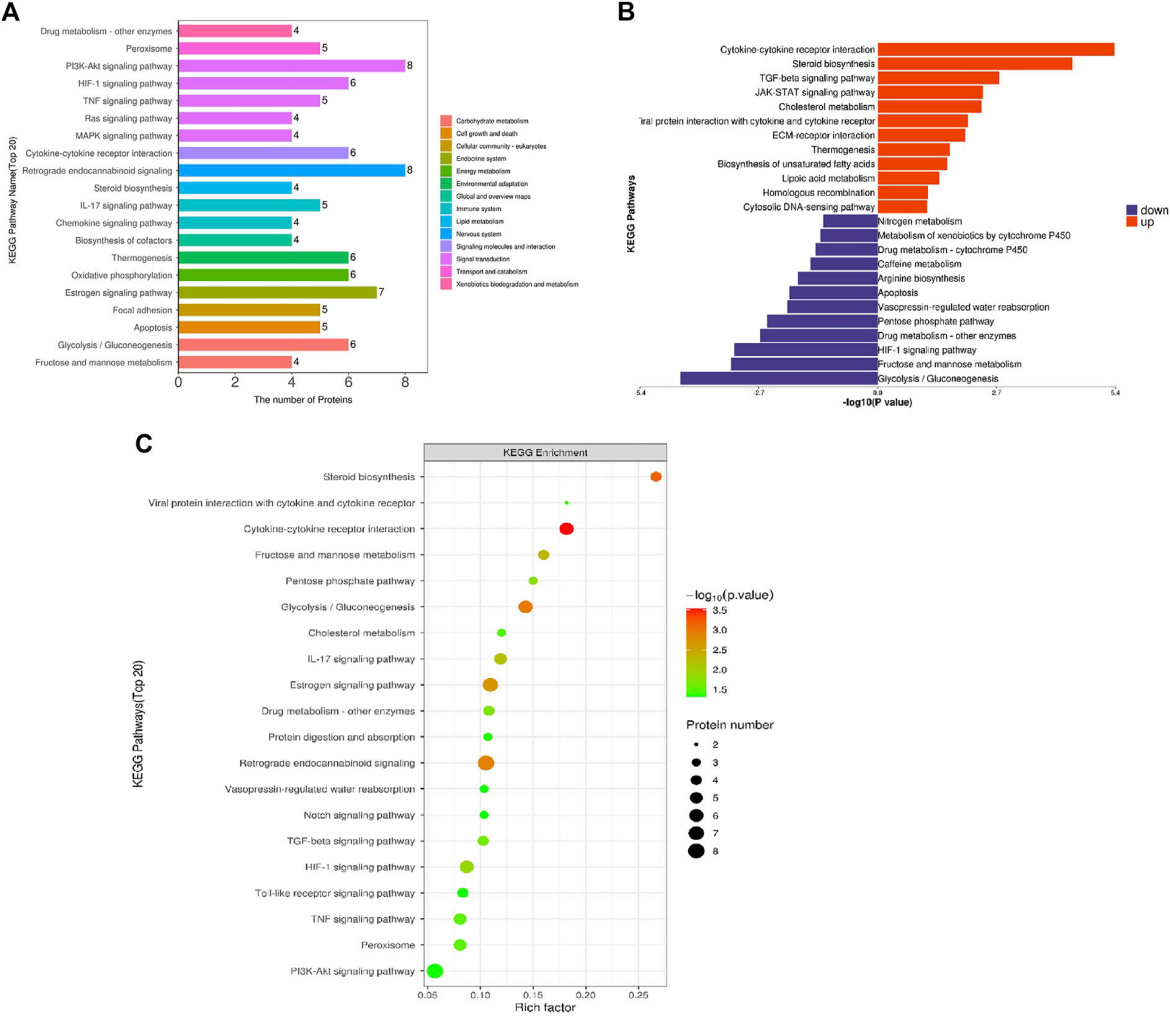
KEGG pathway enrichment analysis of differentially expressed proteins in loperamide-treated HT22 cells. **(A)** KEGG pathway annotation statistics for differentially expressed proteins. **(B)** KEGG pathway differential expression analysis map. **(C)** KEGG pathway enrichment bubble map.

To systematically and comprehensively analyze the biological processes, the mechanisms of disease occurrence, and the mechanisms of drug action, it is often necessary to explain the changes in protein levels from the perspective of protein coordination, such as changes in metabolic pathways. Therefore, proteins were annotated using the KEGG pathway database to analyze the cellular pathways affected by loperamide (Figure 3). We found that loperamide treatment significantly changed protein levels in the PI3K-Akt signaling, retrograde endocannabinoid signaling, estrogen signaling, glycolysis/gluconeogenesis, oxidative phosphorylation of AMPK and PPAR signaling pathways (Figure 3A). Specifically, proteins in the cytokine-cytokine receptor interaction, JAK-STAT signaling, and TGF-beta signaling pathways were upregulated and glycolysis/gluconeogenesis, HIF−1 signaling, fructose, and mannose metabolism were downregulated (Figure 3B). These results were obtained using Fisher’s Exact Test (*p*-value < .05).

**FIGURE 4:**
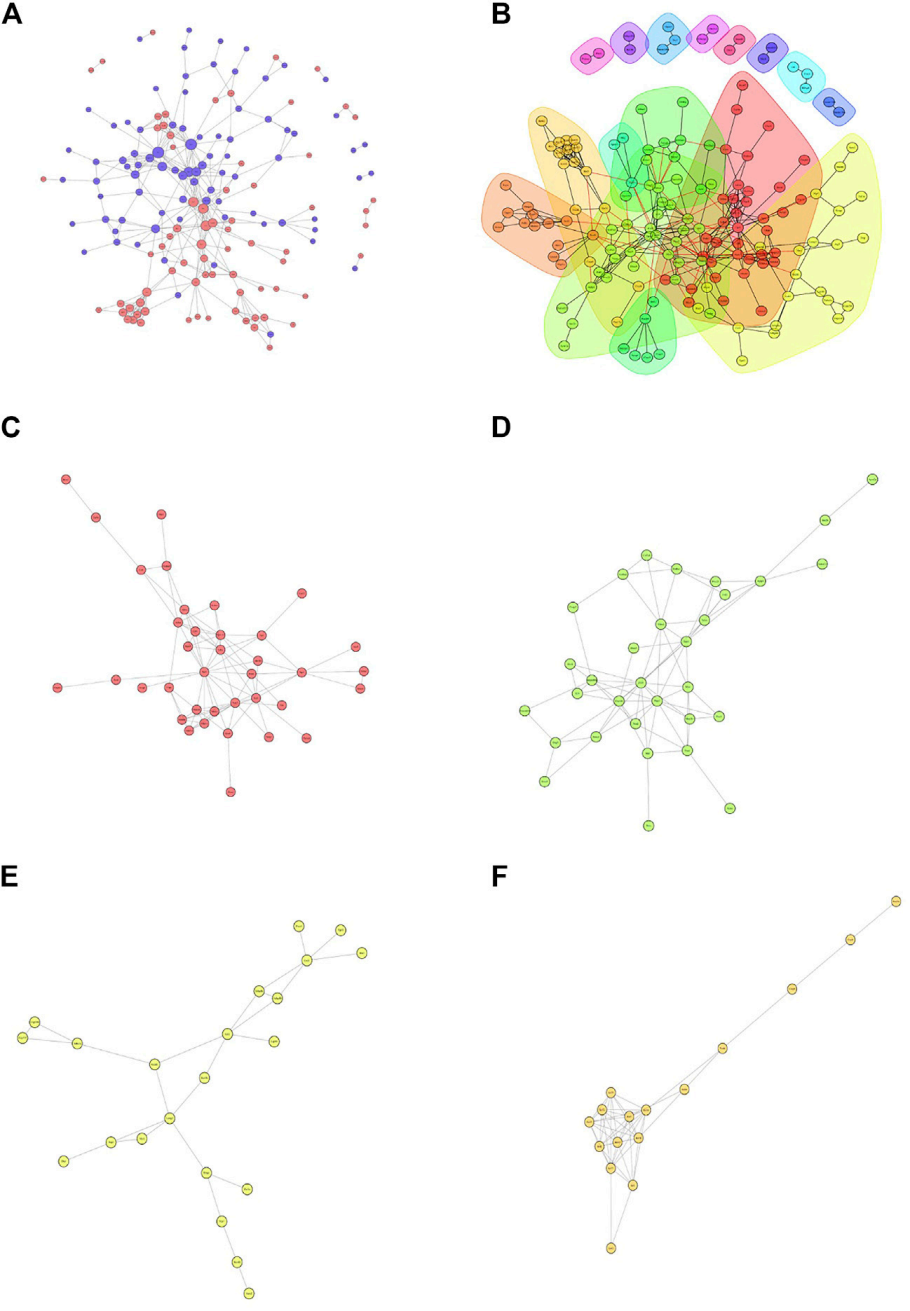
Analyses of the protein-protein interaction networks for the differentially expressed proteins in loperamide-treated. **(A)** Group differentially expressed protein interaction network diagram. **(B)** Interaction network diagram for functional classification diagram **(C–F)** interaction network of key genes diagram.

Then, we constructed a protein-protein interaction (PPI) network of the differentially expressed proteins detected following loperamide therapy using Cytoscape software, which is based on protein interaction links in the STRING or IntAct databases (Figure 4A). In the PPI interaction network, highly aggregated proteins often have the same or similar functions and synergistically exert their biological functions. Therefore, based on the principle of topology identification, the highly aggregated proteins in the interaction network were divided into different clusters (Figure 4B). Concurrently, we incorporated correlation analysis and identified the interaction map of key genes affected by loperamide (Figures 4C–F).

### Identification of gene expression altered after loperamide treatment by RT-PCR

**FIGURE 5:**
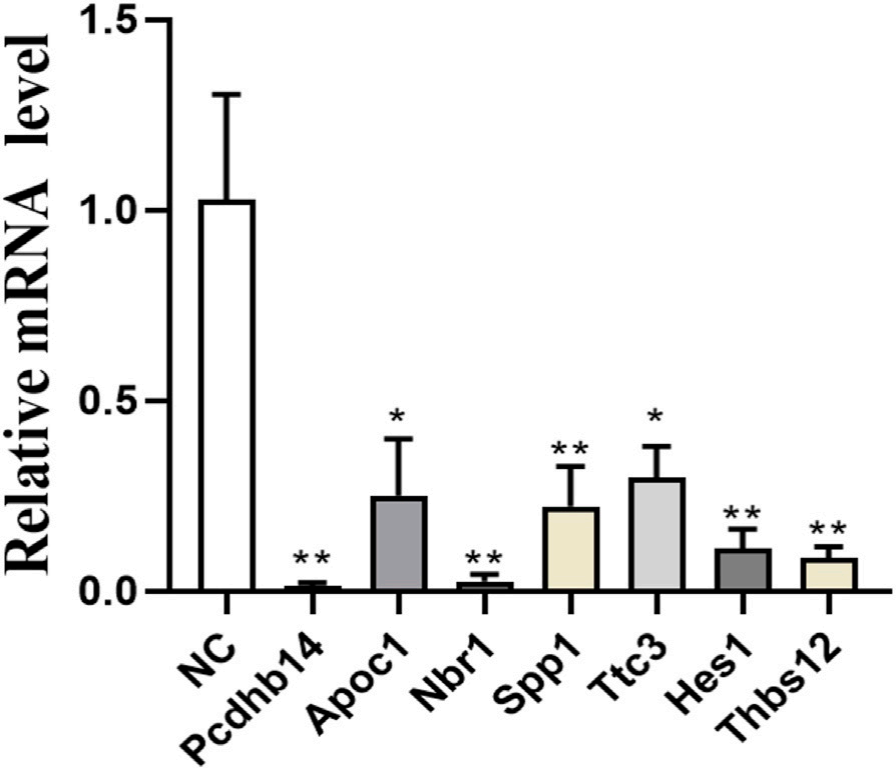
Expression of Pcdhb14, Apoc1, Nbr1, Spp1, Ttc3, Hes1, Thbs1 genes after loperamide treatment by QPCR (*p < 0.05; **p < 0.01).

Based on the results of proteomic sequencing, we analyzed differentially expressed genes. Combined with relevant literature analysis, we determined that loperamide treatment altered the expression of *Pcdhb14*, *Apoc1*, *Nbr1*, *Spp1*, *Ttc3*, *Hes1*, *Thbs1* genes, using RT-PCR analysis (Figure 5).

## Discussion

Loperamide is a potent mu opioid agonist which acts as an adjunctive analgesic for oral pain caused by mucositis or cancer (Regnard et al., 2011). Although loperamide is clinically safe, its anesthetic effect, on neuronal cells, is unknown. In this study, we comprehensively compared the proteome of control and loperamide-treated HT22 cells using an LC-MS/MS-based method and identified 7,820 proteins. Comprehensive bioinformatics analysis showed that loperamide treatment affected numerous signaling pathways, including PI3K-Akt, tumor necrosis factor (TNF), and Notch. Based on proteome sequencing and RT-PCR validation, our results show that mu opioid receptor activation can affect the expression of related genes in neural cells, including *Pcdhb14*, *Apoc1*, *Nbr1*, *Spp1*, *Ttc3*, and *Hes1*, and is neurotoxic to HT22 cells. Our findings provide guidance for clinical anesthesia.

Protocadherin Beta 14 (PCDHB14) is a calcium-dependent cell adhesion protein. Protocadherins are a large family of molecules expressed in the central nervous system and have been implicated in the formation and maintenance of synaptic connections. Several mental functions, including learning and memory, are dependent on it, including neuronal survival and axonal projection. Studies demonstrate that EtOH alters the expression of adhesion molecules, neuronal migration, and neurite outgrowth, hence impairing neuronal development (Liesi, 1997; Vangipuram et al., 2008). EtOH treatment has been reported to decrease *Pcdhb14* expression during neuronal differentiation (Halder et al., 2015); Thus, in line with other research, our results imply that loperamide-induced downregulation of this protocadherin gene may affect synaptic function and hinder the development of the central nervous system (CNS).

Apolipoprotein CI (APOC1) is a candidate Alzheimer’s disease (AD) gene, and variants of the gene, such as rs11568822, are associated with AD risk and may be functionally related to AD pathophysiology. A key pathological feature of AD is the production of neuritic plaques, intracellular formation of neurofibrillary tangles, and neuronal loss through extracellular deposition of amyloid beta (Aβ) peptide (Ca Ccamo and Oddo, 2012). Accumulation of mutant APP (mAPP) and Aβ could lead to neuronal dysfunction (Reddy et al., 2018), and mitophagy enhancers resist effectively mAPP and Aβ-induced mitochondrial and synaptic toxicities in AD (Kshirsagar et al., 2022).Apolipoprotein E (APOE) and APOC1 work together to engage in a number of biological processes, including cholesterol metabolism, membrane remodeling, neuronal death, and neuron reconfiguration (Leduc et al., 2010). Accumulating evidence from clinical and pathological studies suggests that the persistent deterioration of cerebral cholesterol metabolism disorders is associated with AD pathophysiology (Leduc et al., 2010; Vestergaard et al., 2010). According to research, frontotemporal dementia and primary progressive aphasia are both caused by APOC1. In Chinese individuals with late-onset Alzheimer’s disease, APOC1 H2 may work in concert with APOE4 to raise the risk of cognitive deterioration. Therefore, mu opioid receptor activation appears to influence lipid metabolism in nerve cells.

The eukaryotic kingdom, NBR1 (the neighbor of BRCA1 gene 1) is a widely expressed and highly conserved protein (Yamamoto et al., 2000; Zu et al., 2004; Mardakheh et al., 2009). Only a few ATG8 family members have recently been shown to interact with NBR1, including gamma-aminobutyric acid type A receptor-associated protein (GABARAP), GABARAP-like 1 (GABARAPL1), and 16 kDa Golgi-associated ATPase enhancer (GATE-16 or GABARAPL2) (Zellner et al., 2021). Importantly, the ubiquitin-associated ubiquitin domain of NBR1 interacts with ubiquitin (Walinda et al., 2014). These characteristics have led to the hypothesis that NBR1 might serve as a cargo adapter for the autophagic degradation of ubiquitinated substrates like p62, which can also bind to members of the ATG8 family of proteins (Zhou et al., 2013). Recently, LC3, GABARAP, and GATE-16 have been reported in Parkinson’s disease (PD) and dementia with Lewy bodies (DLBs) (Kim et al., 2021). NBR1 may have a role in the development of cytoplasmic inclusions in synucleinopathies because it has been found to localize in Lewy bodies (LBs) in PD and DLB as well as glial cytoplasmic inclusions (GCIs) in multiple system atrophy (MSA) In addition, NBR1 has attracted interest because of its location close to *BRCA1* in the genome. NBR1 may function by interacting with calcium and integrin binding (CIB) and fasciculation and elongation protein zeta-1 (FEZ1) proteins in cellular signaling pathways, and its developmentally-restricted expression suggests a possible role in neurodevelopment (Kirkin et al., 2009). Therefore, activation of mu opioid receptors in HT22 cells may influence autophagy in HT22 cells.

Secreted phosphoprotein 1 (SPP1), also known as osteopontin (OPN) is a multifunctional secreted glycoprotein that affects the adhesion, proliferation, differentiation, migration, and survival of multiple cell types (Jessen and Mirsky, 1999; Stoll and Jander, 1999; Vargas and Barres, 2007; Pegoraro et al., 2011). In the peripheral nervous system, SPP1 was identified as a novel SC-expressed gene product regulated by axon-derived signaling (Mantuano et al., 2008). SPP1 was found to be upregulated after sciatic nerve transaction and is responsible for motor regeneration in rats (Wang et al., 2011). However, another study reported that the expression of SPP1 was downregulated after sciatic nerve injury in rats (Love and Thompson, 1998). SPP1 promotes Schwann cell (SC) proliferation and inhibits apoptosis through the PKCα signaling pathway by binding to CD44 andαvβ3 (Pegoraro et al., 2011). Thus, it is likely that mu opioid agonist-treated HT22 cells affect their migration and differentiation.

A physiological component of the cell-autonomous pathway of neuronal development is E3 ubiquitin ligase (TTC3).and its knockdown results in increased neurite extension. However, reductions in TTC3 levels are not sufficient to differentiate neurons and alter Golgi organization (Zhou et al., 2021). TTC3’s primary role may be to suppress neurite outgrowth by acting on growth cones rather than the Golgi apparatus, which is consistent with the finding that only a tiny portion of TTC3 is linked to the Golgi apparatus. However, TTC3 can express itself above physiological levels in Down’s syndrome (DS) and other neurological diseases (Berto et al., 2007), it can disrupt Golgi compactness and impair neurite extension. These findings may be relevant for understanding how elevated TTC3 levels in DS affect the overall intellectual disability phenotype. Mu opioid receptor activation in HT22 cells can affect the expression of TTC3, which may affect the extension of neural synapses and affect the development of a neural function.

The transcription factor Hes1 is a key component of the Notch signaling pathway. The transmembrane protein Notch undergoes membrane-region processing upon activation of Notch signaling, releasing the intracellular domain of Notch. The important Notch signaling mediator Rbpj and this fragment subsequently form a complex in the nucleus, where it stimulates the production of downstream genes including Hes1 and Hes5 (Artavanis-Tsakonas et al., 1999; Hiroshi, 2008). Hes1 and Hes5 suppress the expression of proneural genes and prevent neuronal differentiation (Hiroshi, 2008). Thus, through Notch signaling, differentiating neurons prevent nearby cells from maturing into neurons, retaining neural stem cells (NSCs), a process known as lateral inhibition (Artavanis-Tsakonas et al., 1999; Hiroshi, 2008). Rbpj deletion disrupts Notch signaling, impairing lateral inhibition, and depleting both active and quiescent NSCs (Imayoshi et al., 2010). Therefore, loperamide will likely cause the differentiation of neuronal cells HT22 to be affected. There are five members of the thrombospondin (Thbs) stromal cell glycoprotein family (Thbs1–5), and although they are secreted, as intracellular chaperones, they also respond to stress in the endoplasmic reticulum (ER), increase secretory pathway activity, extracellular matrix production, and attachment complex membrane residency (Vanhoutte et al., 2016; Brody et al., 2018; Schips et al., 2019). Thbs proteins from group A form trimers made up of Thbs1 and Thbs2, whereas proteins from group B form pentamers made up of Thbs3, Thbs4, and Thbs5 (Adams and Lawler, 2011). During development, Thbs genes show distinct spatiotemporal expression patterns, but the expression of all five genes after an injury is typically observed only in adults. Single Thbs-null mice have been described as having multiple phenotypes, although our recent discovery that mice with knockout of all five Thbs genes survive into adulthood suggests that these proteins are involved in injury and stress responses. Thbs1 has been demonstrated to induce lethal heart atrophy through PERK-ATF4-regulated autophagy, and so mu opioid receptor agonism may have a role in the regulation of autophagy in HT22 cells.

The phosphatidylinositol-3-kinase (PI3K)/Akt and mammalian target of rapamycin (mTOR) signaling pathways are critical for many aspects of cell growth and survival under physiological and pathological conditions. Protein kinase B (Akt) is also activated by PI3K activation, and Akt then activates mTOR by phosphorylating and deactivating the tuberous sclerosis complex. Cell development in response to advantageous nutrients and other growth stimuli is principally regulated by mTOR. By phosphorylating and inactivating the tuberous sclerosis complex, protein kinase B (Akt) is also activated in response to PI3K activation. Akt then activates mTOR. While mTOR primarily modulates cell growth in response to growth-promoting foods and other stimuli, it also affects aging and other physiological processes connected to nutrition, such as protein synthesis, ribosome biogenesis, and cell proliferation in adults with very restricted growth (Jacinto and Hall, 2003). mTOR has been linked to numerous neurodevelopmental and neuropsychiatric disorders and is essential for the central nervous system’s (CNS) neuronal plasticity, learning, and memory (Costa-Mattioli and Monteggia, 2013). Brain-derived neurotrophic factor (BDNF)-dependent survival requires mTOR activation (Smith et al., 2014). Following CNS damage, conditional deletion of intrinsically negative mTOR regulators such phosphatase and tensin homolog (PTEN) or tuberous sclerosis complex 1 promotes axonal regeneration (Park et al., 2008). Furthermore, activation of mTOR promotes changes in dendritic morphology and the formation of synaptic contacts (Urbanska et al., 2012). Memory reconsolidation and late long-term potentiation (L-LTP) depend on mtor-mediated translational control (Stoica et al., 2011). Our results suggest that mu-opioid receptor agonist treatment of HT22 cells mainly affects the PI3K/AKT signaling pathway, and thus the PI3K/AKT signaling pathway may play an important role as a major regulatory mechanism for the pathological state of HT22 cells. And some studies have also shown that the PI3K/AKT signaling pathway is necessary for mu-opioid receptor activation. Our proteomic sequencing results are consistent with previous studies (Kam et al., 2004).

In summary, we found that mu opioid receptor activation may affect the PI3K/AKT signaling pathway, Notch signaling pathway, neuronal apoptosis, differentiation, autophagy, and lipid metabolism in neurons, Especially the PI3K/AKT signaling pathway. This may be part of the mechanism of the well-known toxic effects of mu opioid receptor activation on nerve cells during the process of medication. In conclusion, our study guides clinical anesthesia and advances our understanding of mu opioid receptor agonist-induced nerve damage. In the future, it will be of interest to combine mu opioid receptor agonists and inhibitors to further study the function and role of mu opioid receptors in nerve cells.

## Conclusion

We used mu opioid receptor agonists to study the effect of mu opioid receptor activation on nerve cells. Through proteomic sequencing and RT-PCR, we examined the expression of relevant target genes after mu opioid receptor activation and correlated the results with observed nerve cell damage.

## Data Availability

The original contributions presented in the study are publicly available. This data can be found here: http://proteomecentral.proteomexchange.org/cgi/GetDataset/ PXD004687.
